# Associations between depression and gingivitis among adolescents resident in semi-urban South-West Nigeria

**DOI:** 10.1186/s12903-021-01421-6

**Published:** 2021-02-08

**Authors:** Morenike Oluwatoyin Folayan, Maha El Tantawi, Nneka Maureen Chukwumah, Michael Alade, Boladale Mapayi, Olakunle Oginni, Olaniyi Arowolo, Nadia A. Sam-Agudu

**Affiliations:** 1 Faculty of Dentistry, Obafemi Awolowo University, Ile-Ife, Osun State Nigeria; 2 Faculty of Dentistry, Alexandria University, Alexandria, Egypt; 3 School of Dentistry, University of Benin, Edo State, Nigeria; 4 Obafemi Awolowo University Teaching Hospitals Complex, Ile-Ife, Nigeria; 5 Department of Mental Health, Obafemi Awolowo University, Ile-Ife, Osun State Nigeria; 6 International Research Center of Excellence, Institute of Human Virology Nigeria, Abuja, Federal Capital Territory Nigeria; 7 Institute of Human Virology and Department of Pediatrics, University of Maryland School of Medicine, Baltimore, USA

**Keywords:** Adolescents, Gingivitis, Depression, Oral health, Mental health, Nigeria

## Abstract

**Objective:**

None of the past studies that had showed a linked between oral and mental health among adolescents was conducted in Nigeria. The objective of this study was to determine the association between gingivitis and depression among adolescents in Ile-Ife, South-West Nigeria.

**Methods:**

This cross-sectional study collected data through a household survey conducted between December 2018 and January 2019. Adolescents aged 10 to 19 years old were identified using multistage sampling. The study outcome measure was gingivitis, measured by the Löe and Silness gingival index. The explanatory variable was depression, measured by the Patient Health Questionnair. Confounders considered were age, sex, socioeconomic status, frequency of daily tooth brushing, oral hygiene status (measured by the plaque index), consumption of refined carbohydrates in-between meals, use of dental floss, and history of dental service utilization in the past 12 months. A logistic regression model was constructed to determine risk indicators for moderate/severe gingivitis. Additionally, modification of associations between dependent variables and the significant risk indicators of depression was assessed.

**Results:**

Mean plaque index for the 1,087 adolescent participants enrolled in the study was 0.80. We found a prevalence of 8.5% for moderate/severe gingivitis and 7.9% for depression. In adjusted regression, there were significant associations between the presence of moderate/severe gingivitis and consumption of refined carbohydrates in-between meals (OR 1.94, 95% CI 1.14, 3.28) and plaque index (OR 16.56, 95% CI 10.03, 27.33). Depression also significantly modified the association between plaque index and the presence of moderate/severe gingivitis (*P* < 0.0001), with a stronger association observed with mild depression (OR 24.75, 95% CI 3.33, 184.00) compared with no depression (OR 15.47, 95% CI 9.31, 25.69), with no significant modification for the association with frequent consumption of refined carbohydrates (*P* = 0.06).

**Conclusion:**

Although there was no significant association between gingivitis and depression among our adolescent Nigerian cohort, depression significantly modified the association between plaque index score and moderate/severe gingivitis.

## Background

Periodontal disease is a pandemic that can cause general health disorders and reduce quality of life [1]. Periodontitis induces chronic systemic inflammation, which stimulates the production of pro-inflammatory cytokines, thereby contributing to an increase in neuro-inflammatory response [2–6]. This response is associated with the initiation and/or progression of several systemic conditions, such as cardiovascular disease [7], chronic obstructive pulmonary disease [8], chronic kidney disease [9] and diabetes [10]. Periodontal infection induces inflammation that may increase the risk of tumor-promoting effects [11, 12], and is associated with malignancies of the lung [8], kidney [13], pancreas [10], esophagus [11], and blood/bone marrow [14]. Periodontal infection is also associated with adverse pregnancy outcomes [15], atherosclerosis [16], rheumatoid arthritis [17] and neurodegenerative conditions such as Alzheimer’s disease [18, 19].

Periodontal diseases are common among adolescents [20], and when not managed or poorly managed, may start a life-course of systemic diseases. It is associated with depression [21], a disorder whose onset is often in early adolescence [22]. Depression is also associated with neglect of oral care, due to reduced motivation and interest [23]. The increased cigarette smoking and alcohol dependence observed in depressed patients is also associated with periodontal diseases [24, 25]. Furthermore, depression can modify the immune response, and can also increase the risk of affected individuals engaging in unhealthy oral health habits, thereby increasing the risk for periodontium diseases [26].

While dentists and dental care specialists are able to detect periodontal disease during clinical evaluations, non-dental medical practitioners are much less likely to evaluate or diagnose periodontal diseases [27]. Identifying health symptoms that are indicative of periodontal diseases may help increase the index of suspicion among medical practitioners, thereby potentially intercepting a lifetime risk of systematic diseases. This study aimed to determine the association, if any, between depression and gingivitis among adolescents in a semi-urban community in South-West Nigeria. Specifically, the study evaluated for associations between gingivitis and depressive symptoms, hypothesizing that there was an association between the two health conditions.

## Methods

### Study design, setting, and population

Data was collected through a household survey conducted between December 2018 and January 2019 in Ife Central Local Government Area of Osun State, a semi-urban community in South-West Nigeria. Adolescents were eligible for the study if they were 10 to 19-years old and living in the study setting. Adolescents who were mentally challenged, critically ill and who were otherwise unable to respond independently to the survey were excluded from participation. Written individual consent, and assent and/or parental consent were obtained per national guidelines (See Ethical Considerations). Recruitment of participants continued until study sample size was reached.

### Sample size and sampling technique

The minimum sample size was calculated with the formula proposed by Araoye [28]. With a caries prevalence of 13.9% among 12-year-olds in the study setting [29], a margin of error of 5%, and a confidence level of 95%, the minimum sample size was 1323 adolescents. Adolescents were recruited using a multi-stage sampling technique. First, 70 of the 700 enumeration areas in Ife Central Local Government Area were sampled with a simple random technique. Next, every other household in the selected enumeration areas was identified as an eligible household. Finally, in each household, one adolescent who met the inclusion criteria was recruited for study participation. The next eligible household was substituted whenever a household declined to participate.

### Socioeconomic status

Data on socioeconomic status were collected with an adapted version of the index developed by Olusanya et al. [30], which had been used in a previous study in the same setting [31]. This index is a multiple-item index combining the mother’s level of education with the father’s occupation. For this study, data were collected on the educational levels and professions of respondents’ parents. The social class of the adolescent was determined by adding the score of the mother’s level of education to that of the father’s occupation. Each adolescent was allocated into social classes I–V (class I: upper class; class II: upper middle class; class III: middle class; class IV: lower middle class; and class V: lower class). When an adolescent had lost a parent, the socioeconomic status was determined using the status of the living parent. When an adolescent had lost both parents, the socioeconomic status was determined using the status of the caregiver/guardian.

### Tooth brushing

Respondents were also asked to indicate the frequency of tooth brushing using the following alternatives—irregularly or never, once a week, a few (2–3) times a week; once a day, and more than once a day. Respondents who chose the options ‘irregularly or never, once a week, a few (2–3) times a week; once a day’ were classified as not having undertaken preventive dental care [32].

### Use of dental floss

Respondents were also asked to indicate how often dental floss was used for to clean the teeth using the following alternatives—‘Not at all, occasionally, a few (2–3) times a week, once in a day, more than one time in a day’. Respondents, who chose the options ‘Not at all, occasionally, a few (2–3) times a week’, were classified as not having undertaken preventive dental care [32].

### Consumption of refined carbohydrate in-between-meals

Respondents were also asked to indicate the frequency of consuming sugar-containing snacks or drinks between main meals using the following alternatives—‘About 3 times a day or more, about twice a day, about once a day, occasionally; not every day, rarely or never eat between meals.’ Respondents who chose the options ‘About 3 times a day or more, about twice a day, about once a day’, were classified as not having undertaken preventive dental care [32].

### Dental service utilization

Respondents were also asked to indicate the time of the last check-up using the following alternatives—‘within the last 6 months, more than 6 months to one year ago, more than 1 to 2 years ago, more than 2 to 5 years ago, more than 5 years, never, do not remember.’ Attending a dental check-up within the last year was defined as preventive care use. Respondents who chose the options ‘more than 1 to 2 years ago, more than 2 to 5 years ago, more than 5 years, never, do not remember’ were classified as not having undertaken preventive dental care [32].

### Depression symptoms

The Patient Health Questionnaire (PHQ-9) is a nine-item questionnaire with scores for each of the nine DSM-IV criteria, from "0" (not at all) to "3" (nearly every day). The PHQ-9 can be used to monitor the severity of depression and response to treatment. It has 61% sensitivity and 94% specificity in adults [33]. Possible scores range from 0 to 27. Scores 0–4 indicate no depression, 5–9: mild depression, 10–14: moderate depression, 15–19: moderately severe depression, and 20–27: severe depression [33]. It has good concurrent validity with Beck’s depression inventory (0.61) and has a one-month test–retest reliability of 0.89 among young Nigerian adults [34].

### Oral hygiene

Each participant was examined sitting, under natural light, with sterile dental mirrors by trained dentists. The teeth were examined wet. Plaque index [35] was used to determine oral hygiene status. The plaque index score was based on six numerical determinations representing the amount of debris found on the surfaces of index permanent teeth 12, 16, 24, 32, 36, and 44. The mesial, distal, buccal, and lingual gingival areas of the index teeth are scored from 0 (no plaques) to 3 (abundance of soft matter within the gingival pocket and/or on the tooth and gingival margin). The mean score for each tooth is obtained, and the mean score for the individual is determined by adding the indices for each tooth and dividing by the number of teeth examined.

### Gingival health

The presence and severity of gingivitis was evaluated with the gingival index, described by Löe and Silness [36]. Changes in the gingiva in relation to the appropriate six index teeth (16, 12, 24, 36, 32 and 44) in the permanent dentition were assessed. Four areas of each index tooth were scored, and the scores were summed and divided by four to give the gingival index for each tooth. The gingival index of each participant was obtained by adding the values of all index teeth and dividing by six. Gingivitis was classified as mild, moderate, or severe, with values of 0.1–1, 1.1–2, and 2.1–3, respectively. Gingivitis was dichotomized into normal gingiva/mild gingivitis and moderate/severe gingivitis [36].

### Data analyses

Descriptive analyses were conducted to determine the proportion of adolescents with the study variables. Bivariate analyses were conducted to determine the associations between the explanatory variables (depressive symptoms), confounders and the outcome variable (gingivitis) using chi square and t tests. Univariate logistic regression models were constructed to determine the association between moderate/severe gingivitis and the explanatory and confounding variables. Two multivariable models were constructed: one including individual, behavioral and clinical factors, and the other including these factors in addition to depressive symptoms. Using separate multivariable models, we also assessed modification by depressive symptoms of the associations between the presence of mild to severe gingivitis and variables significantly associated with it in bivariate analyses. The estimated coefficients, expressed as adjusted odds ratios (AOR) and their 95% confidence intervals, were calculated. The statistical analyses were conducted using SPSS for Windows version 23.0 (IBM Corp., Armonk, N.Y., USA). Statistical significance was inferred at *P* ≤ 0.05.

### Ethical considerations

Ethical approval was obtained from the Ethics and Research Committee of the Institute of Public Health, Obafemi Awolowo University, Ile-Ife, Nigeria. Approval for conduct of the study was obtained from the Local Government Authority prior to commencement. The study was conducted in line with guidance from the Federal Ministry of Health [37]. Efforts were made to minimize risks and loss of confidentiality by ensuring anonymized data collection was conducted privately and collected with an electronic data platform. Study participants’ discomfort with the personal nature of questions was limited by ensuring field workers were trained on how to ask sensitive questions and to clarify non-verbal cues observed during the interviews. No compensation was paid for study participation.

## Results

Table 1 shows the sociodemographic profile of 1087 study participants who had complete data for this study. Most adolescents in the study were males (609, 56.0%) with average age of 14.7 years. The majority of study participants brushed their teeth less than once a day (90.8%), did not floss daily (87.9%), consumed refined carbohydrates in between meals more than once daily (59.3%) and had not visited the dentist in the 12 months preceding the study (98.7%). There was similar distribution of study participants by high (33.3%), middle (34.6%) and low (32.1%) socioeconomic status. Their mean plaque index was 0.80 and 7.9% had various levels of depression. Half of the participants had mild gingivitis (547, 50.3%), 87 (8%) had moderate gingivitis and 5 (0.5%) had severe gingivitis, while 448 (41.2%) had no gingivitis.

**Table 1:** Sociodemographic profile, oral health behaviors, and depressive symptoms of 10–19-year-old adolescents in Ile-Ife, Nigeria and their association with the presence of moderate to severe gingivitis [N = 1087]

Variables		Whole sample n (%)	Normal/mild gingivitis n (%)	Moderate/severe gingivitis n (%)	*P* value
Age	Mean (SD)	14.7 (2.7)	14.65 (2.67)	14.66 (2.65)	0.97
Sex	Male	609 (56.0)	554 (55.7)	55 (59.8)	0.45
	Female	478 (44.0)	441 (44.3)	37 (40.2)	
Socioeconomic status	High	400 (36.8)	371 (37.3)	29 (31.5)	0.49
	Middle	357 (32.8)	326 (32.8)	31 (33.7)	
	Low	330 (30.4)	298 (29.9)	32 (34.8)	
Toothbrushing	< 2 times a day	987 (90.8)	904 (90.9)	83 (90.2)	0.84
	> 2 times a day or more	100 (9.2)	91 (9.1)	9 (9.8)	
Daily use of dental floss	Yes	132 (12.1)	116 (11.7)	16 (17.4)	0.11
	No	955 (87.9)	879 (88.3)	76 (82.6)	
Consumption of refined carbohydrates in between meals	Less than once daily	442 (40.7)	395 (39.7)	47 (51.1)	0.03
	More than once daily	645 (59.3)	600 (60.3)	45 (48.9)	
Utilization of dental services in the last 12 months	Yes	14 (1.3)	11 (1.1)	3 (3.3)	0.11
	No	1073 (98.7)	984 (98.9)	89 (96.7)	
Plaque index score	Mean plaque index score (SD)	0.80 (0.57)	0.72 (0.52)	1.60 (0.54)	< 0.0001
Patient’s health (depression)	Normal	1001 (92.1)	919 (92.4)	82 (89.1)	0.46
	Mild	65 (6.0)	57 (5.7)	8 (8.7)	
	Moderate	14 (1.3)	12 (1.2)	2 (2.2)	
	Moderately severe to severe depression	7 (0.7)	7 (0.7)	0 (0.0)	

Table 1 also displays outcomes of the bivariate analysis. There was a significant association between the presence of moderate or severe gingivitis, plaque index score (*P* < 0.0001) and depression (*P* = 0.04). A higher proportion of adolescents with moderate to severe gingivitis had higher plaque index compared to those with normal gingiva or mild gingivitis (1.57 vs 0.77; *P* < 0.0001). Also, a significantly greater percentage of adolescents with normal gingiva and mild gingivitis were not depressed, compared to those with moderate and severe gingivitis (91.9% vs 85.6%; *P* = 0.04).

In univariate regression (Table 2) and in multivariable regression (Table 2, Model 1) where depression was not included, the presence of moderate/severe gingivitis was significantly associated with frequent consumption of refined carbohydrates in between meals and plaque index. Also, in univariate regression, adolescents with mild depression had a significant two-fold odds of moderate/severe gingivitis compared to adolescents with no depression (OR 2.05, 95% CI 1.12, 3.73). The variables in Model 1 explained 37% of the variation in the presence of moderate/severe gingivitis and those in Model 2, where depression was added, explained almost the same amount of variation as Model 1 (Nagelkerke’s R2 = 0.38).

**Table 2:** Association between the presence of moderate to severe gingivitis and sociodemographic background, oral behaviors and depression in logistic regression analysis [N = 1087]

Factors	Univariate analyses	Multivariate analyses	
	UOR (95% CI)	Model 1 AOR (95% CI)	Model 2 AOR (95% CI)
Age	1.00 (0.92, 1.09)	1.01 (0.91, 1.12)	1.00 (0.90, 1.11)
Male versus female	1.18 (0.77, 1.83)	1.20 (0.72, 2.02)	1.22 (0.73, 2.05)
Twice daily or more tooth brushing versus less than twice daily tooth brushing	1.08 (0.52, 2.22)	0.78 (0.32, 1.89)	0.75 (0.31, 1.83)
Daily use of dental floss versus non-daily use of dental floss	1.60 (0.90, 2.83)	1.15 (0.56, 2.34)	1.20 (0.59, 2.45)
Consumption of refined carbohydrates in between meals more than once daily versus less than that	1.59 (1.03, 2.43)*	1.95 (1.16, 3.29)*	1.94 (1.14, 3.28)*
Utilization dental services in the last 12 months versus no utilization of dental services in the last 12 months	3.02 (0.83, 11.01)	1.67 (0.34, 8.12)	1.73 (0.36, 8.36)
High socioeconomic status versus low socioeconomic status	0.73 (0.43, 1.23)	0.80 (0.41, 1.53)	0.78 (0.40, 1.51)
Middle socioeconomic status versus low socioeconomic status	0.89 (0.53, 1.49)	0.93 (0.50, 1.74)	0.94 (0.51, 1.75)
Plaque index	16.18 (9.94, 26.34)*	16.42 (9.98, 27.03)*	16.56 (10.03, 27.33)*
Mild depression versus normal	2.05 (1.12, 3.73)*	–	1.70 (0.68, 4.25)
Moderate depression versus normal	2.20 (0.74, 6.52)		2.89 (0.51, 16.49)
Moderately severe to severe depression versus normal	0 (0, –)		0 (0, –)
Nagelkerke R2	–	0.37	0.38

UOR, unadjusted odds ratio; AOR, adjusted odds ratio; CI, confidence interval; *: statistically significant at *P* < 0.05

Model 1: adjusted model, excluding depression, Model 2: adjusted model, including depression

In Model 2, which was fully adjusted and included depression, the association between the presence of moderate/severe gingivitis and frequent consumption of refined carbohydrates in-between meals was statistically significant: those with frequent consumption had about two folds the odds of moderate/severe gingivitis than those with less than once daily consumption (OR 1.94, 95% CI 1.14, 3.28). Moderate/severe gingivitis was also significantly associated with plaque index: one-unit higher plaque index score was associated with about 17 folds the odds of moderate/severe gingivitis (OR 16.56, 95% CI 10.03, 27.33). Adolescents with mild or moderate depression had higher odds of moderate/severe gingivitis compared to those with no depression although this difference was not statistically significant (OR 1.70; 95% CI 0.68, 4.25 and OR 2.89, 95% CI 0.51, 16.49).

Table 3 shows that the depression significantly modified the association between plaque index and the presence of moderate/severe gingivitis (*P* < 0.0001) with stronger association with mild (OR 24.75, 95% CI 3.33, 184.00) than no depression (OR 15.47, 95% CI 9.31, 25.69). Depression did not significantly modify the association between the presence of moderate/severe gingivitis and frequent consumption of refined carbohydrates in between meals (*P* = 0.06) although the association among those with mild depression (OR 3.58, 95% CI 0.67, 19.25) was stronger than among those with no depression (OR 1.53, 95% CI 0.97, 2.40).

**Table 3:** Modification of the association between the presence of moderate/severe gingivitis and consumption of carbohydrates and plaques by depressive symptoms [N = 1087]

Association of moderate/severe gingivitis with:	OR (95% CI)				*P* of interaction
	Normal	Mild depression	Moderate depression	Moderately severe to severe depression^a^	
Frequent consumption of refined carbohydrates	1.53 (0.97, 2.40)	3.58 (0.67, 19.25)	0 (0, −)	–	0.06
Plaque	15.47 (9.31, 25.69)*	24.75 (3.33, 184.00)*	4.30 × 10^4^ (0, −)	–	< 0.0001

OR, odds ratio; CI, confidence interval;

^*^Statistically significant at *P* < 0.05

^a^Among adolescents with moderately severe to severe depression, all participants had no to mild gingivitis and therefore no estimates could be generated

## Discussion

The study findings showed that depression significantly modified the association between plaque accumulation and the presence of more severe forms of gingivitis, with stronger association observed among adolescents with more severe depression. Also, there was a strong association between frequent consumption of refined carbohydrates in-between meals and moderate/severe gingivitis in adolescents, after controlling for socio-demographic factors and oral health behaviors; depression did not significantly modify this association, although the association was stronger for those with mild depression than those with no depression.

This study provides empirical evidence on association between mental and oral health among adolescents in Nigeria. The data was collected through a household survey and the sample size was large and adequately powered the study. Our findings can therefore be generalizable to the study community and potentially similar settings within and outside of Nigeria. The study is however based on a cross-sectional design, and thus the associations observed cannot be inferred as a causal relationship. Also, because of the age range of the participants (10–19 years), it was not possible to universally assess periodontal parameters, since it is recommended not to assess pocket depth or clinical attachment loss in patients less than 15 years of age, due to the possibility of pseudo pockets associated with gingival changes in this period [38]. In addition, due to the low prevalence of the more severe forms of depression among adolescents in our cohort, the estimates of association may have limited precision, indicated by large or non-estimable confidence intervals. However, these estimates suggest a possible role for depression on its own or as an effect modifier for the association between risk indicators and gingivitis.

There are multiple suggestions on the route through which depression may influence the association between plaque and gingivitis. First, depression could modify the individual immune response [39, 40]. The depression-modified immune system may also lead to increased pro-inflammatory cytokine production and induction of vascular damage [41]. Individuals who are depressed may also neglect their oral hygiene due to reduced motivation and interest [23] and may increase smoking and alcohol consumption [24, 25]. These factors all increase the risk for periodontal diseases.

The study found no association between the frequency of tooth brushing and moderate/severe gingivitis but did find an association between plaque accumulation and moderate/severe gingivitis. We also found an association between higher frequency of consumption of refined carbohydrates in-between-meals and moderate/severe periodontitis; a relationship non-significantly moderated by mild depression. The risk for moderate/severe periodontitis through plaque accumulation may therefore be moderated by sugar consumption rather than tooth brushing frequency, in our adolescent study population. Refined carbohydrate is a risk factor for periodontal disease [42] and also a risk factor for depression [43–45]. Further studies are needed to explore these postulated relationships between gingivitis, plaque accumulation, sugar consumption and depression.

Our study found a difference between adolescents with no and mild depression in the presence of moderate/severe gingivitis but no difference among those with more severe depression. Other studies conducted outside of Africa have reported otherwise—in Europe [46], North America [47], and Asia [48]. These regions have a higher prevalence of adults who smoke, compared to our younger study population and our geographic study community [49] In studies reporting these associations, it therefore may be possible that increased smoking resulting from depression may have potentiated the association between depression and gingivitis, since they are also risk factors for periodontal diseases themselves [50]. In addition, the present study might have been less able to detect significant associations, given the relatively low prevalence of moderate/severe depression in our cohort. This observed phenomenon needs to be further studied and the plausible explanation for these findings explored further.

## Conclusion

In conclusion, mild depression was associated with greater odds of moderate/severe gingivitis in the study population and it was a modifying factor for the association with plaque accumulation and consumption of refined carbohydrates. The specific pathway for this relationship needs to be explored further through a causal pathway study. Further studies should also take age, geography, and degree of smoking or alcohol consumption into consideration.

## Data Availability

All data generated for this study are presented in the manuscript. Patient-level data can however be accessible on reasonable request from the corresponding author, Morenike Oluwatoyin Folayan.
